# Non-Invasive Ventilation Reduces Postoperative Respiratory Failure in Patients Undergoing Bariatric Surgery: A Retrospective Analysis

**DOI:** 10.3390/medicina59081457

**Published:** 2023-08-12

**Authors:** Francesco Imperatore, Fabrizio Gritti, Rossella Esposito, Claudia del Giudice, Chiara Cafora, Francesco Pennacchio, Francesco Maglione, Antonio Catauro, Maria Caterina Pace, Ludovico Docimo, Claudio Gambardella

**Affiliations:** 1 Unit of Anaesthesia and Intensive Care, “San Giovanni di Dio Hospital” Frattamaggiore, 80020 Naples, Italy; 2 Unit of Intensive Care, Department of Emergency, “A. Cardarelli Hospital”, 80131 Naples, Italy; fabrizio.gritti@gmail.com (F.G.); rossella_48@hotmail.it (R.E.); chiara.cafora@gmail.com (C.C.); 3 Unit of Anaesthesia and Intensive Care, University of Campania “L. Vanvitelli”, 80131 Naples, Italy; mariacaterina.pace@unicampania.it; 4 Division of General, Mininvasive, Oncologic and Bariatric Surgery, University of Campania “L. Vanvitelli”, 80131 Naples, Italy; cdg@libero.it (C.d.G.); francesco.pennacchio@gmail.com (F.P.); francesco.maglione@gmail.com (F.M.); antonio.catauro@unicampania.it (A.C.); ludovico.docimo73@gmail.com (L.D.)

**Keywords:** non-invasive ventilation, bariatric surgery, respiratory failure, Venturi mask, post-anaesthesia care unit

## Abstract

*Background and Objectives*: Postoperative non-invasive ventilation (NIV) has been proposed as an attractive strategy to reduce morbidity in obese subjects undergoing general anaesthesia. The increased body mass index (BMI) correlates with loss of perioperative functional residual capacity, expiratory reserve volume, and total lung capacity. The aim of the current study is to evaluate the efficacy of NIV in a post-anaesthesia care unit (PACU) in reducing post-extubation acute respiratory failure (ARF) after biliointestinal bypass (BIBP) in obese patients. *Materials and Methods*: A retrospective analysis was conducted from January 2019 to December 2020 to compare acute respiratory failure within the first 72 postoperative hours and oximetry values of obese patients who underwent BIBP after postoperative NIV adoption or conventional Venturi mask. *Results*: In total, 50 patients who received NIV postoperative protocol and 57 patients who received conventional Venturi mask ventilation were included in the study. After 120 min in PACU pH, pCO_2_, pO_2_, and SpO_2_ were better in the NIV group vs. control group (*p* < 0.001). Seventy-two hours postoperatively, one patient (2%) in the NIV group vs. seven patients (12.2%) in the control group developed acute respiratory failure. Therefore, conventional Venturi mask ventilation resulted in being significantly associated (*p* < 0.05) with postoperative ARF with an RR of 0.51 (IC 0.27–0.96). *Conclusions*: After bariatric surgery, short-term NIV during PACU observation promotes a more rapid recovery of postoperative lung function and oxygenation in obese patients, reducing the necessity for critical care in the days following surgery. Therefore, as day-case surgery becomes more advocated even for morbid obesity, it might be considered a necessary procedure.

## 1. Introduction

Post-extubation acute respiratory failure (ARF) refers to a condition where a patient experiences respiratory distress or failure, hypoxia, and hypercapnia shortly after being removed from mechanical ventilation (extubation) [1]. Post-extubation acute respiratory failure after a surgical procedure is a challenging and relatively common complication. Several factors can contribute to this complication, such as the effects of anaesthesia, surgical site effects, atelectasis, impaired lung function, fluid shifts and imbalances, airway obstruction, and infections. The management of post-extubation acute respiratory failure involves identifying the underlying cause and providing appropriate interventions. This may include re-intubation and mechanical ventilation if necessary, administering medications to improve respiratory function, addressing airway obstructions, providing respiratory support such as non-invasive positive pressure ventilation, optimising fluid balance, and treating underlying infections or other medical conditions [2].

Postoperative non-invasive ventilation (NIV), in fact, has been proposed as an attractive strategy to reduce morbidity and improve postoperative outcomes in obese subjects undergoing general anaesthesia. Bariatric patients present peculiar negative features: the increased body mass index (BMI) correlates with loss of perioperative functional residual capacity (FRC), expiratory reserve volume (ERV), and total lung capacity (TLC), which decreased by up to 50% of the preoperative values [1]. Obesity hypoventilation might be affected by fat distribution, with predominant upper-body (chest and abdomen) fat distribution being associated with more severe derangements in lung volumes [2]. Therefore, for the abdomen impedance to the diaphragmatic movement, FRC decreases particularly in the supine position. These changes promote atelectasis, determining significant pulmonary ventilation perfusion defects, hypoxemia, poor gas exchange, and consequent hypoventilation. The abovementioned alterations of the respiratory physiology in obese patients can lead to the development of acute and chronic respiratory failure, sleep-related breathing disorders, and postoperative pulmonary complications [3].

In addition to obesity, aesthetic drugs, surgery, and postoperative pain can precipitate these alterations mainly through the reduction in lung volume and the formation of lung atelectasis. Limited studies have proposed different methods capable of attenuating these postoperative ventilatory compromises, such as NIV applied as continuous positive airway pressure ventilation (CPAP), bilevel positive airway pressure (BiPAP), or pressure support (PS) with positive end-expiratory pressure (PEEP), postoperatively after tracheal extubation [4]. In detail, NIV provides ventilatory support to restore and maintain lung volumes by recruiting atelectatic lung regions, allowing for improved oxygenation and reduced work of breathing [5]. However, definitive results about the best treatment to prevent postoperative respiratory failure in patients undergoing bariatric surgery are still lacking in the literature. As NIV is questionable in patients with upper gastrointestinal sutures (e.g., Sleeve Gastrectomy, Roux-en-Y Gastric Bypass), we directed our attention to its application in patients who have undergone biliointestinal bypass (BIBP), in which the anastomoses are performed distally at the jejunum [6].

The aim of the current retrospective analysis is to evaluate the efficacy of NIV in a post-anaesthesia care unit (PACU) in reducing post-extubation acute respiratory failure after BIBP in obese adult patients.

## 2. Materials and Methods

### 2.1. Study Design

This study is reported according to the Strengthening the Reporting of Observational Studies in Epidemiology (STROBE) statement for cohort studies [7]. A retrospective analysis was conducted to evaluate the effectiveness of NIV in a PACU in reducing post-extubation acute respiratory failure after BIBP in obese adult patients. The study adhered to the ethical principles outlined in the Declaration of Helsinki, and written informed consent was obtained from all participants.

### 2.2. Study Setting and Study Population

This study was conducted from January 2019 to December 2020 at the Academic Hospital University of Campania “Luigi Vanvitelli” (Naples, Italy). Inclusion criteria were morbid obesity, defined as body mass index (BMI) > 40 kg/m^2^, and age between 25 and 50 years old, or a BMI > 35 hg/m^2^ and the presence of obesity-related comorbidities, including type 2 mellitus diabetes (T2MD), hypertension, hyperlipidaemia, bronchial asthma, osteoarthritis, and degenerative joint disease, in compliance with the International Federation for Surgery of Obesity guidelines, addressed to BIBP. Patients requiring rapid sequence induction for emergency surgery, suspicious of difficult airway conditions or pre-existing lung impairment, pregnancy, asthma, severe or end-stage renal dysfunction, patients affected by cardiac disease with severe limitation of physical activity (NYHA class > II), and patients affected by severe psychiatric disorders or difficulties in cooperating during measurements were excluded from the study. 

Those affected by morbid obesity underwent elective BIBP. All patients were informed about the NIV technique at the preoperative anaesthesiologic assessment. All the anaesthesia were performed by an experienced team, skilled in the management of bariatric patients (over 200 anaesthesia in obese patients). All surgeries were performed by experienced bariatric surgeons (over 500 bariatric procedures). Preoperative evaluation included anthropometric measurements (height in cm, weight in kg, BMI in kg/m^2^), comorbidities evaluation (HbA1c, C-peptide, stimulated C-peptide, ECG, echocardiography, lower limbs colour-Doppler, thyroid profile), and upper gastrointestinal endoscopy with Helicobacter Pylori assessment and chest X-ray. Before surgery, each patient was assessed for physical signs of difficult mask ventilation and intubation (BMI > 25 kg/m^2^, age > 55 years, jaw protrusion severely limited, lack of teeth, snoring, beard Mallampati class III or IV) using the STOP-BANG questionnaire, which demonstrates the presence of predictive factors of obstructive sleep apnoea (OSA). A STOP-BANG value of 5 or higher is considered a robust predictor of the presence of OSA, and the perioperative identification allows for the risk of postoperative OSA to be minimised (i.e., sit up, avoid opioids, apply oxygen, CPAP treatment as soon as possible) [8].

### 2.3. General Anaesthesia

In all patients, pre-oxygenation started for 3 min with an adjusted fraction of inspired oxygen (FiO_2_) of 1.0. Subsequently, the anaesthesia was induced with fentanyl 2–3 μg/kg and propofol 2 mg/kg, followed by maintenance using a TIVA protocol: remifentanil (0.1–0.2 μg/kg ideal body weight) and propofol 3–6 mg/kg × h. After a single dose of rocuronium (0.5 mg kg−1 ideal body weight), conventional orotracheal intubation was performed without the use of an additional neuromuscular blocking agent. All patients received a reversal of neuromuscular blockade such as Sugammadex at a dose of 4 mg/kg. Standard monitoring (pulse oximetry, non-invasive blood pressure, and electrocardiography) was performed in all cases associated with the monitoring of aesthetic depth levels. Volume-controlled mechanical ventilation was used with a Drager ventilator (Draeger Evita XL, Lubecca, Germany). All patients had TV 6 mL/kg, PEEP 5, RR 14, and FiO_2_ 50%. Fifteen minutes before extubation, each patient received ondansetron (4 mg i.v.) and dexamethasone (4 mg i.v.) as postoperative nausea and vomiting (PONV) prophylaxis, and ketorolac (10 mg i.v.) and Tramadol (50 mg i.v.) for postoperative pain management. The peripheral nerve stimulator by train-of-four (TOF) was adopted to monitor the neuromuscular blockade. Before extubation, in all cases, the oral cavity was suctioned. Extubation was performed once the patient was fully awake and spontaneously breathing. SpO_2_ at awakening was always recorded. Thereafter, the patients were transported to the PACU. Following extubation, the aforementioned drug regimen was repeated within the first 24 h as follows: Ketorolac 10 mg i.v. every 8 h (total of 30 mg) and tramadol 50 mg i.v. every 8 h (total of 150 mg) for pain control. In addition to the PONV prophylaxis administered prior to extubation, Metoclopramide 10 mg i.v. was repeated every 8 h (total of 30 mg) for the initial 24 h post-extubation in case of persistent nausea or vomiting. This regimen was applied to both groups of patients under continuous medical supervision and monitoring.

### 2.4. Post-Anaesthesia Treatments

Patients were assigned to either the non-invasive ventilation group (NIV group) or the conventional Venturi mask ventilation group (control group). In the control group, patients were provided with a Venturi mask set at an FiO_2_ of 60% and a flow rate of 15 L/min. Conversely, patients assigned to the NIV group underwent a 120 min cycle of PSV plus PEEP while wearing a full-face mask. Ventilation was administered using a Draeger Ventilator, configured with the following fundamental settings: Inspiratory Pressure Difference (DeltaPInsp) of 10 mmHg and PEEP of 5 mmHg, both accompanied by an FiO_2_ of 60%. These settings were adjusted individually for each patient to achieve a tidal volume of approximately 5–6 mL/kg of ideal body weight. The procedure was conducted for 120 min for all patients in both groups.

### 2.5. Outcome Measures

All patients in PACU underwent continuous monitoring for 120 min, which included pulse oximetry, non-invasive blood pressure, and electrocardiography evaluation. In details, a blood gas analysis, using pre-heparinised syringes with puncture in radial artery, was conducted at fixed intervals (0–30–60–90–120 min). This analysis aimed to evaluate SpO_2_, pH, PaO_2_, and PaCO_2_. Acute respiratory failure (ARS) was defined as patient’ respiratory distress accompanied by the recruitment of accessory muscle in association with at least one of the following conditions: arterial oxygen saturation lower than 80%, respiratory acidosis, hemodynamic instability or need for inotropic drugs, and acute decrease in Glasgow coma score (GCS) to less than 7.

The rate of ICU admission within the initial 72 postoperative hours was also recorded.

Postoperative pain levels were assessed using the visual analogue scale (VAS) immediately upon awakening and at 15 min intervals over the span of 120 min. Analgesia was supplemented through intravenous morphine titration, with a maximum dose of 10 mg. All patients were fully awake during this evaluation period.

### 2.6. Study Outcomes

The primary outcome was the evaluation of post-operatory acute respiratory failure rate and oximetry values (SpO_2_, pH, pO_2_, pCO_2_) of obese patients who underwent BIBP after postoperative NIV adoption or conventional Venturi mask application.

The secondary outcome was the evaluation of pain according to the VAS scale in patients addressed to NIV adoption or conventional Venturi mask application in the early postoperative period (120 min).

### 2.7. Statistical Analysis

Continuous variables were described using mean and standard deviation if normality assumptions hold true; median and interquartile range were used otherwise. Qualitative variables are presented in frequencies and percentages compared through Χ2 tests. For all tests, the cut-off for statistical significance was set at *p* = 0.05. Statistical analysis was performed using R v3.5.0. In order to evaluate the association between ICU admission and postoperative management (NIV vs. conventional Venturi mask ventilation), an odds ratio (OR) analysis was performed. Continuous variables were described as mean ± standard deviation (SD). 

## 3. Results

Between January 2019 and December 2020, a total of 119 morbid obesity patients referred to our institution to perform BIBP. In total, 12 patients did not meet the inclusion criteria, whereas 107 met the eligibility criteria. Of these, 50 patients received NIV protocol and 57 underwent conventional Venturi mask ventilation.

Baseline characteristics were similar in the two groups and are summarised in Table 1. All patients in both groups presented an American Society of Anesthesiologists (ASA) score of III. The mean STOP-BANG value was not significantly different (*p* = 0.142) in the two groups (3.84 ± 0.70 and 4.06 ± 0.72 in NIV group and control group, respectively). Likewise, the mean Mallampati value was not significantly different (*p* = 0.162) in the two groups [NIV group (2.5 ± 1.1) and control group (2.2 ± 0.9)]. The overall mean operative time was 98 ± 28 min: no statistical difference was found between the two groups [NIV group (119 ± 26) and control group (97 ± 24), (*p* = 0.447)] (Table 1).

### 3.1. Primary Outcome

All patients were ventilated according to the respective target values. No unexpected intubation problems were recorded in either group. Preoperative pulse oxygen saturation (%SpO_2_) levels were similar (*p* = 0.432) and fell within the normal range (97.03 ± 1.85 vs. 97.41 ± 2.01 in NIV group and control group, respectively). No difference between groups before and after premedication was found. 

At the first postoperative assessment in the PACU, %SpO_2_ resulted 94.21 ± 2.03 vs. 93.98 ± 2.13 in the NIV group and control group, respectively (*p* = 0.348) (Table 1). 

After 120 min in PACU, pH resulted 7.44 ± 0.06 vs. 7.21 ± 0.16 (*p* < 0.001), pCO_2_ was 47.30 ± 1.95 vs. 55.25 ± 3.43 (*p* < 0.001), pO_2_ resulted 67.91 ± 3.03 vs. 60.14 ± 4.17 (*p* < 0.001), and SpO_2_ resulted 96.03 ± 1.93 vs. 93.95 ± 2.09 (*p* < 0.001) in the NIV group vs. control group, respectively (Table 2).

Seventy-two hours postoperatively, ARS requiring ICU admission resulted in being statistically lower in the NIV group (*p* < 0.05): one patient (2%) in the NIV group in the ICU vs. seven patients (12.2%) in the control group. Therefore, conventional Venturi mask ventilation resulted in being significantly associated (*p* < 0.05) with postoperative ICU admission with an OR of 0.51 (IC 0.27–0.96).

### 3.2. Secondary Outcome

No patient experienced severe postoperative pain. In detail, at the end of surgery, the VAS value resulted in being 4.9 ± 2.5 and 4.5 ± 2.1 in the NIV group and control group (*p* = 0.452). In the first postoperative hour (0–60 min), the mean VAS value was 4.3 ± 2.1 and 4.1 ± 2.7 in the NIV group and control group (*p* = 0.426). In the subsequent hour (61–120 min), the VAS value decreased to 3.5 ± 2.4 and 3.6 ± 1.9 in the NIV group and control group (*p* = 0.376). During the observation period, every patient had an acceptable level of vigilance.

## 4. Discussion

ARF can occur early in the postoperative course, and approximately 8% to 10% of these patients may necessitate endotracheal intubation and mechanical ventilation. In the case of ARF, NIV is considered an alternative to invasive mechanical ventilation that can reduce the rate of tracheal intubation as well as complications and mortality [9,10].

The results of two randomised controlled trials, analysed within a Cochrane Library Database, showcased the effectiveness of CPAP and NIV in adults with acute respiratory insufficiency after upper abdominal surgery [10]. The utilisation of CPAP and non-invasive positive-pressure ventilation (NPPV) resulted in diminished intubation rates and shortened ICU stays. Additionally, these interventions notably improved blood gases and blood pH within one hour of implementation. Moreover, the interventions also decreased the risk of pneumonia, sepsis, and surgical wound infection. Notably, the use of CPAP and NPPV appears to be safe as it does not result in anastomotic leakages [10].

In the current series, the early use of NIV in obese patients after extubation led to an improvement in blood gas values, a lower risk of respiratory complications, and a decreased use of intensive care after surgery compared to patients who underwent conventional Venturi mask ventilation. To the best of our knowledge, this is the first study analysing NIV outcomes in patients who have undergone BIBP. After 120 min of PACU, in fact, pH (*p* < 0.05), pCO_2_ (*p* < 0.001), pO_2_ (*p* < 0.001), and SpO_2_ (*p* < 0.001) resulted in all being improved in the NIV group vs. control group. Moreover, NIV allowed for the ARF events and ICU admission to be significantly limited (one patient in NIV group vs. seven patients in control group, *p* < 0.05). 

A possible explanation of this improvement might be the adoption of positive pressure ventilation which restores the FRC to preoperative levels, improving postoperative oxygenation and performance [9]. Our findings align with those of Huerta et al. and Chalhoub et al. who adopted NIV in morbidly obese patients undergoing sleeve gastrectomy and open bariatric surgery. They found that patients treated with NIV demonstrated an increase in PaO_2_ during the postoperative period (PaO_2_: 78.87 ± 8.31 in NIV group vs. 64.27 ± 6.33 in oxygen group) [11,12].

Our findings were also similar to those of Ahmad et al., who showed a successfully postoperative use of NIV to correct atelectasis, allowing for the restoration of the FRC, preventing upper airways collapse, and increasing lung compliance [13]. 

General anaesthesia is frequently related to respiratory impairment and atelectasis. The development of atelectasis is primarily attributed to the compression and reabsorption of oxygen resulting from a high fraction of inspired oxygen. This phenomenon becomes evident within minutes. Despite its recognised negative impact on lung function [14,15], a high FiO_2_ is typically administered at the conclusion of general anaesthesia.

Furthermore, morbid obesity is correlated with a substantial increase in chest wall elastance, airway obstruction, and lung derecruitment, thereby elevating the likelihood of atelectasis development compared to non-obese patients [11].

Vassilakopoulos et al. conducted an analysis of respiratory complications following laparotomic bariatric surgery [16]. The study revealed that pneumonia and atelectasis were the most common issues, which is consistent with prior research findings. These complications can be attributed to various factors, including diminished mucociliary clearance, limited bed mobility, decreased secretion, and alterations in the physiological respiratory pattern. These alterations result in shallower and primarily thoracic breathing, leading to a decreased ability to effectively cough and an accumulation of pulmonary secretions. The utilisation of NIV has demonstrated its efficacy in improving respiratory system compliance by reversing pulmonary microatelectasis, reducing respiratory effort, effectively mitigating pulmonary complications, and enhancing gas exchange. The authors also emphasised the central role of respiratory muscle dysfunction in the development of postoperative acute respiratory failure (ARF).

The cause of postoperative muscle dysfunction after upper abdominal surgery remains not entirely comprehended. Several theories suggest that irritation, inflammation, and trauma in proximity to the diaphragm could lead to local mechanical impairment, potentially contributing to dysfunction [16]. However, as of now, there is a dearth of available data to either substantiate or refute this hypothesis. Studies examining the intrinsic contractility of the diaphragm, evaluated through bilateral electrical stimulation of the phrenic nerves, have shown no discernible alterations after upper abdominal surgery. Animal models have indicated that a reflexive inhibition of the diaphragm might play a role in postoperative dysfunction, although there exists a scarcity of direct evidence supporting this theory in humans [17,18]. Nevertheless, postoperative pain appears to be among the triggering factors in muscular dysfunction [16].

Thus, in obese patients, with or without obstructive sleep apnoea (OSA), supplemental oxygen therapy might not be effective in preventing postoperative oxygen desaturation and hypoxic episodes. Ahmad et al. reported that, following bariatric surgery, 33% of obese patients with OSA and 44% of obese patients without OSA experienced more than five hypoxemic episodes per hour [13]. As a result, the perioperative management of obese patients should encompass measures such as postoperative non-invasive ventilation (NIV) to mitigate the risks of extubation failure, postoperative hypoxemia, respiratory complications, prolonged stays in the post-anaesthesia care unit (PACU), or unplanned intensive care unit (ICU) admissions [19].

Based on the current data, short-term NIV which commenced immediately after extubation, during PACU observation, is capable of enhancing pulmonary function in the first 72 postoperative hours. The benefits of NIV in this context are likely linked to its early initiation after surgery, preceding its application in other surgical procedures among normal-weight populations, as reported in several studies. In the current obese population, NIV appears to be particularly effective due to the respiratory changes primarily arising from FRC reduction. These effects are mitigated by NIV ventilation with PEEP, which begins early after extubation, thereby augmenting alveolar ventilation and improving gas exchange. Given that the measurements were taken nearly five minutes after the discontinuation of anaesthesia, respiratory enhancement cannot be solely attributed to oxygen supplementation/NIV. Even postoperative mobilisation does not completely counteract postoperative respiratory changes [20]. The benefits of NIV in bariatric surgery encompass improved oxygenation, a reduction in postoperative complications, and enhanced recovery. Therefore, it can be inferred that short-term NIV is beneficial and exhibits a lasting impact, potentially associated with increased FRC, enhanced alveolar ventilation, and the prevention of atelectasis.

The results of this retrospective analysis could hold significant relevance for the daily clinical practice of a tertiary care hospital, establishing NIV after BIBP as a tangible choice with far-reaching implications for patient outcomes. Its immediate application in bariatric patients upon extubation demonstrated a noteworthy and statistically significant enhancement (*p* < 0.001) in the primary blood gas parameters (i.e., pH, pCO_2_, pO_2_, SpO_2_) when compared to patients subjected to conventional Venturi mask therapy (control group). Furthermore, the current data indicated a reduced need for ICU admissions in the NIV group, an exceptional outcome that potentially ensures a smoother postoperative recovery for bariatric patients and optimises resource utilisation. This approach also helps mitigate potential risks associated with ICU overcrowding, including incidents such as central venous catheter infections, healthcare-related infections, and ventilator-induced pneumonia. Consequently, NIV emerges as a safe and non-invasive technique with a minimal learning curve, poised to enhance the postoperative trajectory for bariatric patients.

However, this study has several limitations that need to be addressed. Firstly, the patient selection process could benefit from refinement. Additionally, NIV was exclusively evaluated in patients undergoing BIBP, raising questions about its applicability and therapeutic benefits in other major surgeries, especially those requiring upper gastrointestinal sutures [21,22,23]. Notably, it should be acknowledged that NIV mandates trained personnel and the acquisition of costly equipment. Furthermore, consensus criteria for its prophylactic use in the immediate postoperative period have not been definitively established. Moreover, this study did not delve into a direct comparison between NIV and CPAP alone. Finally, considering the current observational period, no conclusion about long-term clinical outcome (i.e., pneumonia incidence) can be achieved. 

## 5. Conclusions

In the current series, the early initiation of short-term NIV in obese patients during PACU observation promotes the more rapid recovery of postoperative lung function and oxygenation, reducing the critical care necessity in the days following surgery. Soon after surgery in obese patients, NIV acts on lung volumes when surgical trauma and anaesthesia are most likely to impact pulmonary function. Therefore, as day-case surgery becomes more advocated even for morbid obesity, it might be considered a necessary procedure. Larger comparative studies are required to achieve definitive results.

## Figures and Tables

**Table 1:** Baseline and anaesthesiologic characteristics of patients in NIV group and control group.

	NIV Group (*N* = 50)	Control Group (*N* = 57)	*p*
Age (years)	52 ± 11	53 ± 9	0.447
BMI	38 ± 3.3	39 ± 2.9	0.311
STOP-BANG score	3.84 ± 0.70	4.06 ± 0.72	0.142
Mallampati score	2.5 ± 1.1	2.2 ± 0.9	0.162
Preoperative %SpO_2_	97.03 ± 1.85	97.41 ± 2.01	0.432
Surgery time (min)	119 ± 26	97 ± 24	0.447
Remifentanil consumption (µg)	1315 ± 210	1279 ± 199	0.113
Propofol consumption (mg)	665 ± 122	679 ± 143	0.454
Time to extubation (min)	8.3 ± 4.9	9.2 ± 5.3	0.476
Postoperative %SpO_2_	94.21 ± 2.03	93.98 ± 2.13	0.348

BMI (body mass index).

**Table 2:** pH, pCO_2_, and pO_2_ in arterial blood gas and SpO_2_ at pulse oximetry saturation in NIV and control groups after 120 min in PACU (post-anaesthesia care unit).

	NIV Group (*N* = 50)	Control Group (*N* = 57)	*p*
pH	7.44 ± 0.06	7.21 ± 0.16	<0.001
pCO_2_ (%)	47.30 ± 1.95	55.25 ± 3.43	<0.001
pO_2_ (%)	67.91 ± 3.03	60.14 ± 4.17	<0.001
SpO_2_ (%)	96.03 ± 1.93	93.95 ± 2.09	<0.001

## Data Availability

The datasets used and/or analysed during the current study are available on reasonable request.
